# Effect of captopril on post-infarction remodelling visualized by light sheet microscopy and echocardiography

**DOI:** 10.1038/s41598-021-84812-7

**Published:** 2021-03-04

**Authors:** Urmas Roostalu, Louise Thisted, Jacob Lercke Skytte, Casper Gravesen Salinas, Philip Juhl Pedersen, Jacob Hecksher-Sørensen, Bidda Rolin, Henrik H. Hansen, James G. MacKrell, Robert M. Christie, Niels Vrang, Jacob Jelsing, Nora Elisabeth Zois

**Affiliations:** 1 Gubra, Hørsholm Kongevej 11, B, 2970 Hørsholm, Denmark; 2 Lilly Research Laboratories, Eli Lilly and Company, Indianapolis, IN 46285 USA; 3 Present Address: Novo Nordisk, 2760 Maaloev, Denmark

**Keywords:** Imaging, 3-D reconstruction, Fluorescence imaging, Cardiovascular models, Light-sheet microscopy, Experimental models of disease, Cardiovascular biology, Heart failure, Drug discovery

## Abstract

Angiotensin converting enzyme inhibitors, among them captopril, improve survival following myocardial infarction (MI). The mechanisms of captopril action remain inadequately understood due to its diverse effects on multiple signalling pathways at different time periods following MI. Here we aimed to establish the role of captopril in late-stage post-MI remodelling. Left anterior descending artery (LAD) ligation or sham surgery was carried out in male C57BL/6J mice. Seven days post-surgery LAD ligated mice were allocated to daily vehicle or captopril treatment continued over four weeks. To provide comprehensive characterization of the changes in mouse heart following MI a 3D light sheet imaging method was established together with automated image analysis workflow. The combination of echocardiography and light sheet imaging enabled to assess cardiac function and the underlying morphological changes. We show that delayed captopril treatment does not affect infarct size but prevents left ventricle dilation and hypertrophy, resulting in improved ejection fraction. Quantification of lectin perfused blood vessels showed improved vascular density in the infarct border zone in captopril treated mice in comparison to vehicle dosed control mice. These results validate the applicability of combined echocardiographic and light sheet assessment of drug mode of action in preclinical cardiovascular research.

## Introduction

Although timely primary coronary percutaneous intervention has substantially improved patient survival post myocardial infarction (MI), the often-concomitant cardiac dysfunction and heart failure affect a significant number of patients. Infarction leads to rapid replacement of cardiomyocytes by fibrotic scar tissue and remodelling of both the infarcted area and the viable myocardium are critical determinants of long-term survival. Despite extensive research, pharmacological treatment options to limit adverse cardiac remodelling and restore myocardial function are insufficient and evaluation of new cardioprotective strategies remains an important objective.

Angiotensin converting enzyme inhibitors (ACEi), including captopril, became the first-line therapeutics for acute MI in 1970s and have been consistently shown to improve patient survival and left ventricular (LV) ejection fraction (EF) after acute MI^1–4^. The beneficial effects arise from attenuation of LV dilation, reduction of preload and afterload, improved oxygen supply, systemic neurohormonal changes and altered cytokine environment^5,6^. In early phase following MI ACEi inhibit fibrosis and can thereby have a limiting effect on infarct size^7^. Newer ACEi (i.e. lisinopril, tradolapril, zofenopril) with improved pharmacokinetics and comparable beneficial effects on patient survival have been developed^8^. Better understanding of the dynamic phases in post-MI remodelling and the role the existing drugs play in these can lead to the development of new therapeutics.

Left anterior descending artery (LAD) ligation in mice and rats remains the most common technique in preclinical pharmacological research to assess the efficacy of treatment options for MI^9^. Consistent with clinical findings, chronic captopril treatment attenuated left ventricular dilation and improved long-term survival in rat LAD-ligation model^10,11^. Similarly, captopril has been shown to exert beneficial effects on aortic flow parameters and myocardial strain following LAD ligation in mice, increasing overall survival^12,13^. Since young mice have been shown to survive extensive MI with limited concomitant mortality^12^ it opens up possibilities to characterize the effect of pharmaceuticals in late-stage post-MI remodelling.

Microvascular rarefaction in the infarcted heart limits blood supply to cardiomyocytes and has emerged as a promising therapeutic area of intervention^14–16^. Angiogenesis in the infarcted heart involves the growth of new capillaries from the endocardium and through the border zone, lying at the interface of healthy and infarcted tissue^17,18^. To date, both vascular density and border zone topology have remained challenging to analyse on tissue sections, due to the highly irregular shape and extent of the infarct^19^. Optical clearing of mouse and even human organs and tissues combined with light sheet fluorescence microscopy (LSFM) has opened new possibilities for visualizing cells and activated signalling pathways in 3D^20–24^, but shortage of automated quantitative analysis platforms is still a bottleneck and no studies have so far demonstrated the sensitivity of the method in preclinical pharmacological cardiovascular research. The current study was aimed at developing a light sheet imaging platform for myocardial infarction analysis and to use it for demonstrating the mode of action of delayed captopril treatment.

## Methods

### Experimental design

**Figure 1:**
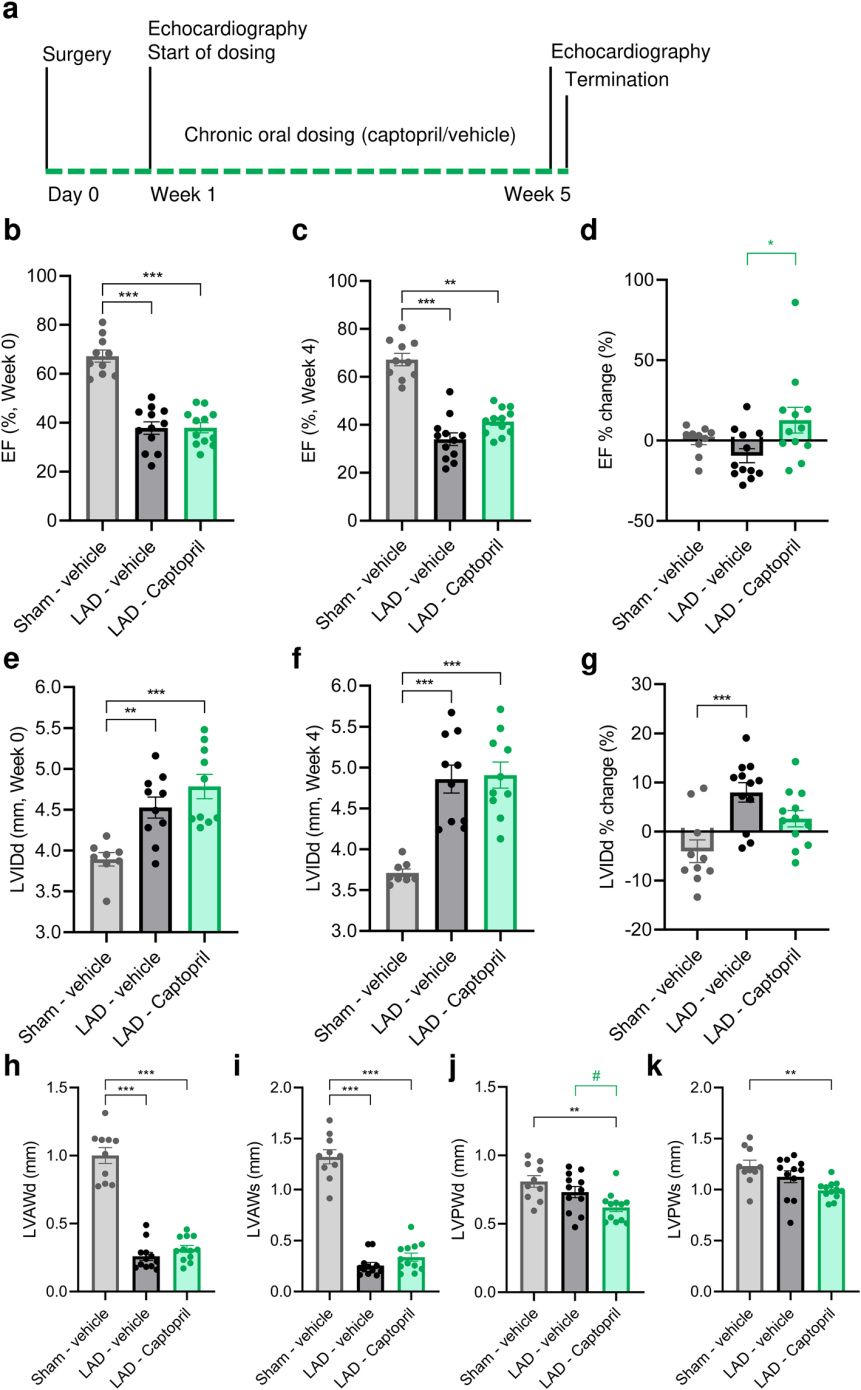
Echocardiographic evaluation of cardiac function and remodelling in a mouse model of myocardial infarction. (**a**) Schematic study outline. (**b**) Ejection fraction (EF) at the time of inclusion in week 0. (**c**) EF after 4 weeks of dosing with either vehicle or captopril. (**d**) EF change over study period (week 4-week 0)/week 4*100%. (**e**) Left ventricle internal diameter in diastole (LVIDd) at the time of inclusion in week 0. (**f**) LVIDd at week 4. (**g**) LVIDd change over study period. (**h**) Left ventricle anterior wall dimension/thickness (LVAW) in diastole and (**i**) systole. (**j**) Left ventricle posterior wall dimension/thickness (LVPW) in diastole and (**k**) systole. Data is presented as mean ± s.e.m. n = 10–12. One-way ANOVA with Tukey’s post hoc test. Significance: *p < 0.05, **p < 0.01, ***p < 0.001. **#:** Significant (p < 0.05) after removal of single non-responder in LAD—Captopril. LAD: left anterior descending artery ligation.

7 days after surgery LAD ligated mice (n = 24) were randomized into treatment groups by both LV internal diameter in diastole (LVIDd) as well as ejection fraction (EF), measured by use of transthoracic echocardiography (Fig. 1a). Enrolment was thus based on both cardiac function as well as remodelling. From the day of enrolment echocardiography, 4 weeks of treatment with either saline (LAD—vehicle, n = 12) or ACEi (LAD—Captopril, n = 12) was initiated. Sham operated mice served as healthy controls (Sham—vehicle, n = 10).

### Animals

Male C57BL/6 J mice (23–25 g, 6 weeks old, Janvier Labs, France, n = 34) were single-housed in a controlled environment (20–22 °C, humidity 40–60%) with a 12 h light/dark cycle. The animals were acclimatized for at least one week before surgery. All animals had ad libitum access to chow diet (Altromin 1324, Brogaarden) and tap water throughout the study period. Body weight and 24-h water intake were measured daily. The studies were approved by The Danish Animal Experiments Inspectorate (license no. 2018–15-0201–01,499), conformed to the European Parliament Directive on the Protection of Animals Used for Scientific Purposes (2010/63/EU) and comply ARRIVE guidelines.

### Ligation of the left anterior descending artery

Before surgery, mice received acetaminophen and buprenorphine analgesia. Surgery was performed under 2% isoflurane as described by Gao et al.^25^. In brief, a small cutaneous incision was made on the left side of the chest wall and the pectoral muscles were bluntly dissected to visualize the fourth intercostal space. A small hole was pierced through the intercostal muscles with a haemostat and the ribs were retracted. With a gentle grip the heart was pushed through the opening and the LAD was identified and ligated using Perma-hand silk suture 7-0 (Ethicon). Subsequently, the heart was quickly returned to the thoracic cavity and the pneumothorax was manually evacuated. Finally, the skin was sutured, and the mouse was allowed to recover. Buprenorphine analgesia was administered for two days after surgery. Sham surgery was performed by exposing the heart and inserting the suture, but without tightening the ligature around the LAD artery.

### Echocardiography

Seven days after LAD ligation or sham surgery, the resultant infarction was assessed by echocardiography using a Vevo 3100 system (VisualSonics, Fujifilm) equipped with a MX400 20–46 MHz linear array transducer (Visual Sonics, Fujifilm). The mice were anesthetized (Isoflurane 2%) and were placed in a supine position on a heating pad and chest hair was removed. Body temperature measured by a rectal probe and heart rate was monitored continuously during the examination. Two-dimensional (2D) parasternal long- and short-axis views were obtained to visualize the location and extent of the MI. Subsequently, 2D-guided M-mode in the parasternal short axis view was used to calculate EF and LV internal diameters in systole and diastole (LVIDs and LVIDd, respectively) using Vevolab software (VisualSonics version 3.2.0, Fujifilm). Only LAD-ligated mice with presence of an anterior wall myocardial infarction extending from the mid-papillary level into the apex were included in the study. EF and LV internal diameters in systole and diastole (LVIDs and LVIDd) were measured by 2D-guided M-mode from the parasternal short axis view and EF was used for randomization into study groups.

After four weeks of treatment EF, LVIDs and LVIDd were re-assessed using the same procedures as described for the enrolment echocardiograph, including placement of the M-mode plane for the evaluation of EF to mimic the plane used during enrolment. Furthermore, LV end-diastolic volume was estimated by tracing the inner perimeter of the LV in parasternal long axis view in end-diastole.

LVID and EF percent change from week 0 was calculated as: (LVID or EF in week 4—LVID or EF in week 0)/ LVID or EF in week 0 × 100%. All echocardiographic examinations and post-hoc analyses were performed by the same examiner blinded to the identity of the mouse.

### Captopril

Captopril (Medchem Express, 0.2 mg/ml) was dissolved in tap water. Cherry flavoured Kool-Aid (Kraft Foods, Inc) was added to the drinking water to increase palatability in captopril as well as vehicle treated mice.

### Tissue processing for light sheet imaging

Mice received a 100 µl tail vein injection of 1 mg/ml Lycopersicon Esculentum (Tomato) lectin conjugated to DyLight 649 (DL-1178, Vector Laboratories, Burlingame, CA, USA) that binds to glycoproteins and reliably labels perfused blood vessels^26^. Mice were placed in isoflurane chamber, maintaining 3.5% isoflurane concentration. After 7 min anaesthesia was confirmed by lack of active paw reflex. While maintaining mice under terminal isoflurane anaesthesia rib cage was opened to expose the heart. Room temperature freshly prepared cardioplegic solution (St. Thomas' Hospital cardioplegic solution number 2: NaCl 110.0 mM, NaHCO3 10.0 mM, KCl 16.0 mM, MgCl2 16.0 mM, CaCl2 1.2 mM; pH 7.8) was injected into the right atrium with a 27G needle to induce diastole and stop the heart. Euthanasia was performed by cutting through the aorta and vena cava and removing the heart. Once removed, the heart was perfused through the aorta using the above described cardioplegic solution to remove fluorescent lectin from cardiac chambers. Hearts were subsequently perfused with freshly prepared cold glyoxal solution for rapid fixation^27^ and immersion-fixed overnight at 4 °C. For tissue clearing, the samples were dehydrated in methanol/water series: 20%, 40%, 60%, 80%, 100%; 1 h each at room temperature. The samples were next transferred to fresh 100% methanol and incubated for two days at room temperature on a horizontal shaker. Next, the samples were incubated by shaking in 66% dicloromethane (DCM)/33% methanol overnight. They were washed twice for 1 h by shaking in 100% DCM at room temperature and taken to dibenzyl ether (DBE). Hearts were weighed after transferring to DBE.

In initial experiments comparing the different fixation conditions the hearts were excised as above, washed in room temperature phosphate buffered saline for 1–2 min (without perfusion and removal of blood) and then immersed in either 4% paraformaldehyde (PFA), 10% neutral buffered formalin (NBF) or glyoxal-based fixative and fixed overnight. The samples were cleared as indicated above.

### Light sheet microscopy

Hearts were imaged using a Lavision ultramicroscope II (Miltenyi Biotec GmbH, Bergisch Gladbach, Germany) with Zyla 4.2PCL10 sCMOS camera (Andor Technology, Belfast, UK), SuperK EXTREME supercontinuum white-light laser EXR-15 (NKT Photonics, Birkerød, Denmark) and MV PLAPO 2XC (Olympus, Tokyo, Japan) objective. Samples were mounted in the same orientation and fixed by the posterior wall with soft neutral silicone gel to a custom-made transparent silicone sample holder. Hearts were imaged in a DBE filled chamber. ImSpector microscope controller software (v7) was used (Miltenyi Biotec GmbH, Bergisch Gladbach, Germany). Whole-heart imaging was carried out at 1.2 × magnification, using 560 ± 20 nm excitation (84% software-controlled laser power cut-off at 100% NKT laser output) and 650 ± 25 nm emission wavelengths at 217 ms exposure time in a z-stack at 15 μm intervals. 9 horizontal focusing steps were merged in contrast-based blending. For whole heart imaging, Olympus dipping cap (10 mm working distance, #201004) was used.

Vasculature imaging was carried out at 4 × magnification, using 630 ± 15 nm excitation wavelength and 680 ± 15 nm emission wavelength (61% laser output filter, 100% NKT laser). Autofluorescence was captured using 560 ± 20 nm excitation (65% software-controlled laser power cut-off at 100% NKT laser output) and 650 ± 25 nm emission wavelengths. 209 ms exposure time and horizontal focusing in 7 steps was used and z-stacks were captured at 7 µm interval. Optically corrected Lavision dipping cap (LV OM DCC20) with 5.7 mm working distance was used for high resolution imaging.

### Image analysis

Bitplane Imaris software (Oxford Instruments Group, Bitplane, Zurich, Switzerland) was used for 3D tissue rendering and for manual segmentation of the LV chamber to generate training dataset for a deep-learning model. For enhanced accuracy, manual segmentation was performed sequentially on two imaging planes (YZ and XY) on a total of 28 hearts, which were augmented in 3D in random orientation to generate 112 volumes for deep learning model development. 100 volumes were used as training set and 12 for validation purposes. U-net was used for deep learning^28^.

LV chamber morphology, volume analyses, vasculature analysis and wall thickness characterization were carried out in Python. Infarction induced changes in the LV apical morphology was characterised using the apical conicity index^29,30^, which measures the ratio of the total area in the apical part of the LV chamber to the fitted cone and hence more precisely detects local dilation that can remain undetected in sphericity and volume analyses. If the heart is fully conical the index would equal to one. Apical geometry was analysed in long axis 2-chamber views of 2D-projections acquired from 3D image stacks. The 2D-projections were performed along the third principal axis of the LV segmentation obtained by the deep-learning model. Diastole was assessed by visual observation of the imaged heart and by setting volume (above 10 mm^3^) and dimension thresholds (long axis length above 4 mm). Hearts that were below these volume and size thresholds were assessed as not fully diastolic and were excluded from statistical analysis. Image analysis was therefore performed on 8 sham—vehicle, 10 LAD—vehicle and 10 LAD—captopril treated mice. Cardiac curvature maps were derived by converting the LV chamber volume to surface mesh and visualizing it in Meshlab^31^. Lower half of the mesh (apical part) was isolated from each mesh to quantify the distribution of curvature across the surface.

For vasculature and wall thickness analysis, pre-segmentation was applied in Imaris to exclude scar tissue and suture material outside the heart. Vessel segmentation was carried out by applying vessel enhancement filter in Python^32^, that was tuned to enhance bright vessels with an approximate lumen diameter below 10 µm. Subsequent thresholding was applied to the vessel-enhanced image to create the final vasculature segmentation. Ventricular wall thickness was estimated locally using a maximal sphere fitting approach^33^. Correlative analysis of vascular density and wall thickness was applied only on the LV wall and excluded the septum and right ventricle.

All statistical analyses were performed in GraphPad Prism (version 8.4.2). Tukey–Kramer test was used for statistical comparison of multiple groups after ANOVA. Pairwise comparison was done using two-tailed t-test. All data are presented as mean values and standard errors of the mean (s.e.m).

## Results

### Delayed post-MI captopril treatment improves cardiac function

First, we wished to establish whether delayed captopril treatment has a beneficial effect on cardiac function. To model MI the LAD was ligated in 6-week-old mice and pharmacological intervention was started 1 week after the surgery, thereby preventing early ACEi activity on cardiac fibroblasts and allowing fibrotic scar formation.

Seven days after sham surgery or LAD ligation, the survival rate was 100% and 63%, respectively. No mortality was observed during the 4 weeks of drug treatment and daily water intake was similar between the groups (Table 1). All study groups exhibited similar body weight 7 days after surgery (Table 1). Systolic dysfunction and cardiac dilation were evident by decreased EF (p < 0.001, LAD—vehicle and LAD—Captopril vs. Sham—vehicle) and increased LVIDd (p < 0.001, LAD—Captopril vs. Sham—vehicle; p < 0.01, LAD—vehicle vs. Sham—vehicle) in LAD ligated animals compared to sham operated mice (Fig. 1b,e).

**Table 1:** Baseline and terminal characteristics of study groups.

	Sham—vehicle n = 10	LAD—vehicle n = 12	LAD—captopril n = 12
Body weight, onset of treatment (day 7) (g)	22.2 ± 0.3	22.6 ± 0.3	22.1 ± 0.2
Heart rate, day 7 (BMP)	488 ± 19	525 ± 12	515 ± 12
Daily water intake (g/day)	7.5 ± 0.2	7.2 ± 0.1	7.2 ± 0.1
Captopril dose (mg/kg/day)	-	-	640 ± 16
Body weight, termination (g)	22.6 ± 0.3	23.1 ± 0.3	22.4 ± 0.3 ^#^
Heart weight, termination (mg)	134 ± 4	159 ± 6 **	138 ± 6 ^#^
Heart weight ratio to tibia length, termination	7.7 ± 0.2	9.1 ± 0.4 *	7.8 ± 0.3 ^#^
Heart rate, week 5 (BPM)	442 ± 18	484 ± 8	464 ± 16
LV chamber volume (mm)	38.5 ± 4.2	69.4 ± 4.7 ***	72.9 ± 5.1

Body weight and echocardiography derived heart rate measured at the onset of treatment (day 7) and/or termination. Heart weight was measured after tissue clearing for light sheet imaging. End-diastolic LV chamber volume was estimated on parasternal long axis view at the time of termination. Data is mean ± s.e.m. One-way ANOVA with Tukey’s post hoc test. **p < 0.01 ***p < 0.001 vs sham—vehicle, #p < 0.05 vs LAD—vehicle.

After 4 weeks, captopril-treated mice had gained slightly less body weight than the LAD -vehicle group (p < 0.05), whereas Sham—vehicle and LAD—vehicle had similar terminal body weight (Table 1). Heart weight (absolute weight and when normalized to tibial length) was increased in LAD—vehicle compared to Sham—vehicle (absolute weight: p < 0.01; normalized: p < 0.05), whereas LAD—Captopril showed a significantly lower heart weight in comparison to LAD—vehicle (p < 0.05). EF was preserved or even improved in the LAD—Captopril group but remained supressed in in LAD—vehicle group 4 weeks after treatment (p < 0.05, Fig. 1c,d). At the time of termination LVIDd was increased in both LAD—vehicle and LAD—Captopril groups (Fig. 1f, p < 0.001), yet when compared to the onset of dosing, LVIDd was increased in LAD—vehicle, but appeared to be preserved in LAD—Captopril mice (Fig. 1g, p < 0.001). EF remained the same throughout the study in Sham—vehicle mice, whereas LVIDd appeared slightly lower 5 weeks after surgery (Fig. 1d,g). Heart rate during echocardiography was similar across the three groups in week 5 (Table 1).

We next used echocardiography to characterize MI-induced changes in the LV wall. In accordance with diastolic dysfunction LV anterior wall (LVAW) thickness was significantly reduced in LAD-ligated groups (Fig. 1h–i). There was a trend towards increased LVAW thickness in captopril treated mice in comparison to vehicle dosed LAD-ligated mice, but this was not significant. The LV posterior wall (LVPW) thickness was reduced in LAD—captopril group in comparison to Sham—vehicle (Fig. 1j–k).

### Light sheet imaging for automated analysis of LV morphology

**Figure 2:**
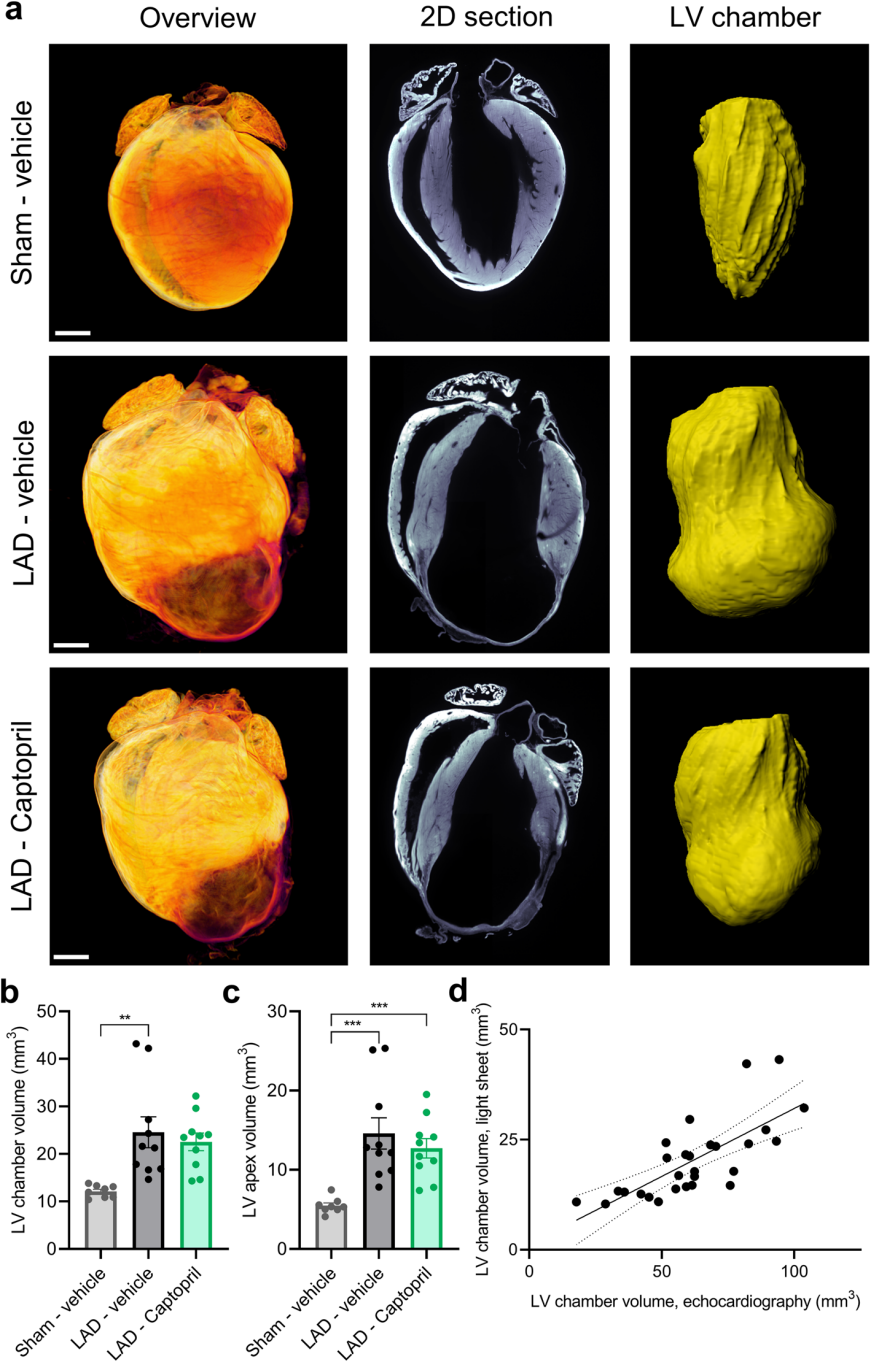
Light sheet imaging and automated analysis of myocardial infarction. (**a**) Representative examples of light sheet imaged hearts. 3D overview image is shown on the left. Second panel from left—digital 2D section is taken from 3D reconstructed heart (long axis horizontal plane). Third panel from left demonstrates deep learning-based segmentation of the left ventricle (LV) chamber (in yellow). (**b**) Diastolic LV chamber volume quantified by deep-learning based analysis. (**c**) LV apex volume (quantified below plane fitted through the center of deep learning segmented chamber). (**d**) Light sheet quantification of LV chamber volume in diastole correlates with echocardiography-based LV end-diastolic volume (r = 0.73, p < 0.0001). Data is presented as mean ± s.e.m., n = 8–10. One-way ANOVA with Tukey’s post hoc test. Significance: **p < 0.01, ***p < 0.001. Scale bars: 1 mm. LAD: left anterior descending artery ligation.

We next established a protocol for rapid fixation and clearing of the heart for light sheet microscopy, with the aim of maintaining high quality cardiac morphology, avoiding tissue degradation, and keeping all analysed hearts in comparable diastolic phase. The choice of the fixative is of key significance in order to maintain correct cardiac morphology (Supplementary Fig. 1a–e). Since intracardiac perfusion fixation damages the heart and fails to keep the hearts in diastolic phase we analysed cardiac morphology in hearts immersion fixed in PFA, NBF and glyoxal, followed by clearing in DBE. This analysis showed that hearts immersion fixed in PFA and NBF suffered from significant irregularities in myocardial morphology as well as from high autofluorescence of blood (Supplementary Fig. 1c,d). In contrast, hearts immersion fixed in glyoxal maintained correct cardiac morphology and had low autofluorescence of blood (Supplementary Fig. 1e). We further optimized the protocol by implementing retrograde perfusion of the excised hearts with cardioplegic solution before glyoxal fixation, thereby enabling to maintain the hearts in diastole for comparable image analysis. The resulting cleared hearts had low uniform autofluorescence, making it feasible to image entire sample at high resolution in approximately 30 min. Light sheet imaging of LAD-ligated hearts provided initial visual overview of the infarct zone due to its thinner ventricular walls and resulting greater optical transparency (Supplementary movies 1 and 2, Fig. 2a).

To enable reproducible analysis of cardiac morphology for drug efficacy studies, a deep learning-based 3D image analysis platform was developed to automatically segment the LV chamber and provide quantitative endpoints on its morphology. Deep learning computational model provided accurate segmentation of chamber volume (Supplementary Fig. 2). Representative images illustrate cross-morphological differences between study groups (Fig. 2a). LAD ligation led to expansion and increase in the LV chamber volume from 12.1 mm^3^ (± 0.5) in the Sham—vehicle group to 24.6 mm^3^ (± 3.2) in LAD—vehicle and 22.5 mm^3^ (± 1.8) in the LAD—Captopril group. The increase in LAD—vehicle group was significant in comparison to Sham—vehicle (p < 0.01) (Fig. 2b). Analysis of the apical part of the LV chamber volume demonstrated a significant increase from 5.46 mm^3^ (± 0.35) in the Sham—vehicle group to 14.58 mm^3^ (± 1.97) and 12.71 mm^3^ (± 1.22) in the LAD—vehicle and LAD—Captopril group, respectively (Fig. 2c). To validate that cleared light sheet imaged hearts maintain their morphological characteristics we compared the LV chamber volume derived from echocardiography with volume calculations from 3D light sheet imaging (Fig. 2d). Tissue clearing results in organ shrinkage, which also reflects in smaller (~ 66%) chamber volume in the light sheet imaged hearts. The reduced heart size is in line with previous data from other cleared organs^34,35^ and permits imaging of entire hearts in single scans. Despite tissue shrinkage there was strong correlation between echocardiography and light sheet measured LV chamber volumes, verifying that heart morphology is maintained throughout the imaging protocol (r = 0.73, p < 0.0001).

**Figure 3:**
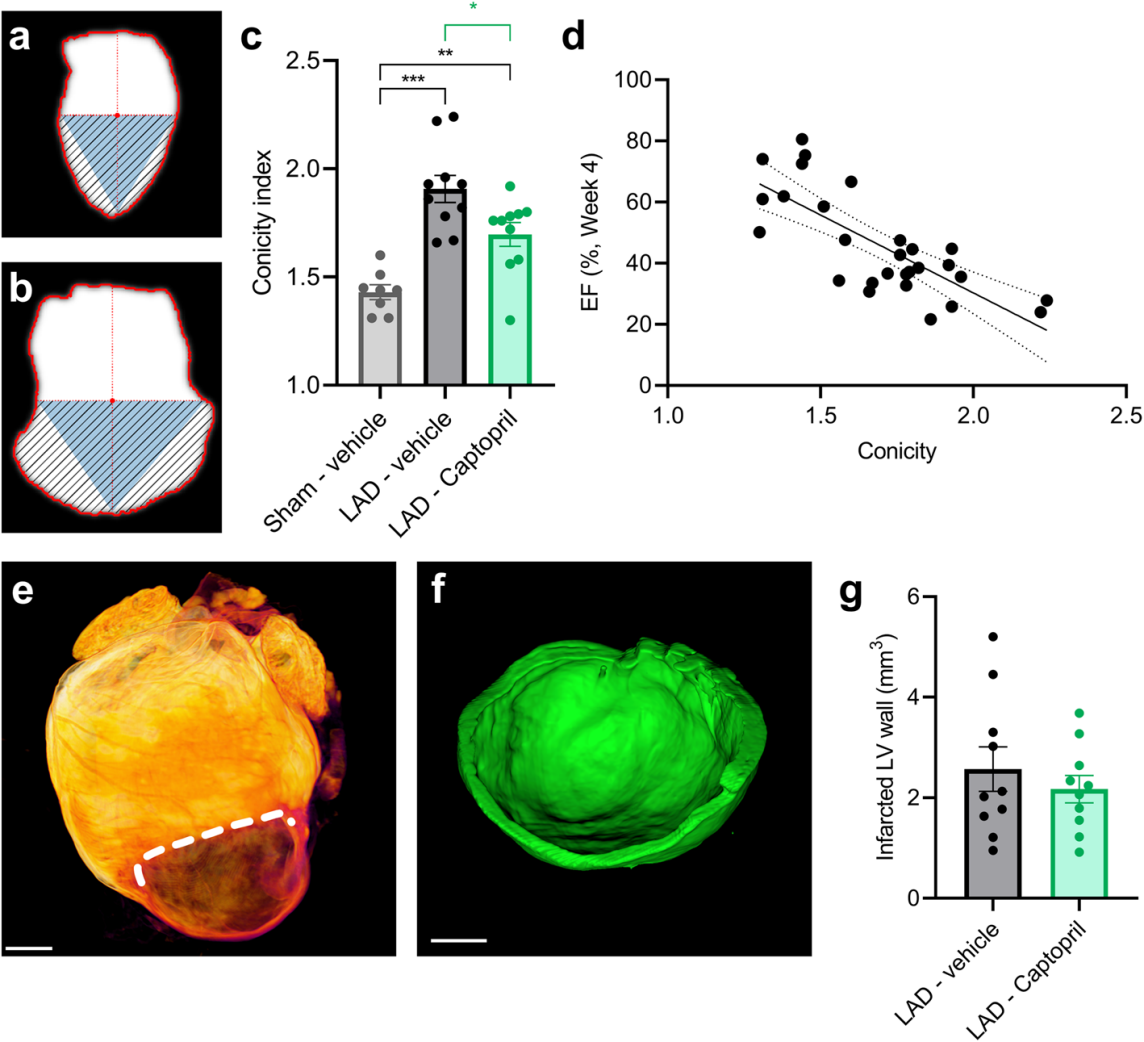
Light sheet analysis of ventricular wall dilation and infarct size. (**a**) Schematic illustration of apical conicity index calculation in Sham—vehicle heart. The index represents the ratio between the fitted triangle and striped area. (**b**) Same as above but shown for LAD—vehicle heart. Left ventricle (LV) wall dilation leads to increased area outside the fitted triangle. (**c**) Statistical comparison of cardiac conicity index. (**d**) Ejection fraction (EF) at week 4 shows negative correlation to conicity index (r = -0.76; p < 0.0001). (**e**) Representative example of light sheet imaged heart with infarct zone separated by line. (**f**) Segmented volume of cardiac wall with thickness below 400 µm (from sample shown in E). (**g**) Quantification of infarcted LV wall volume with thickness below 400 µm. Significance: *p < 0.05, **p < 0.01, ***p < 0.001. Scale bars: 1 mm. LAD: left anterior descending artery ligation.

 We focused next on morphological differences in the infarcted heart to better characterize infarct zone progression. To this end we implemented cardiac conicity index, measuring the dilation of the LV chamber in diastole (Fig. 3a,b). The conicity index showed variability in LAD-ligated hearts. It was for Sham—vehicle 1.43 (± 0.003) and for LAD—vehicle 1.91 (± 0.06) (Fig. 3c). Captopril treatment resulted in improvement in comparison to the LAD—vehicle group, demonstrating a conicity index of 1.70 (± 0.05; p < 0.05) (Fig. 3c). Since LV dilation is expected to result in worse cardiac performance, we compared these results with cardiac EF data and found a strong inverse correlation (r = -0.76, p < 0.0001; Fig. 3d). To compare more precisely the extent of the dilated myocardial wall in the LAD ligated groups we set a threshold of LV wall thickness at 400 µm, as below this consistent loss of cardiomyocytes was evident (Supplementary Fig. 3). Using this approach, the volume of the infarcted LV with thin wall in LAD—vehicle and LAD—Captopril was similar (2.6 mm^3^ ± 0.4 versus 2.2 mm^3^ ± 0.3, Fig. 3e–g) in line with the echocardiography estimates of LVAW mean thickness (Fig. 1h–i).

**Figure 4:**
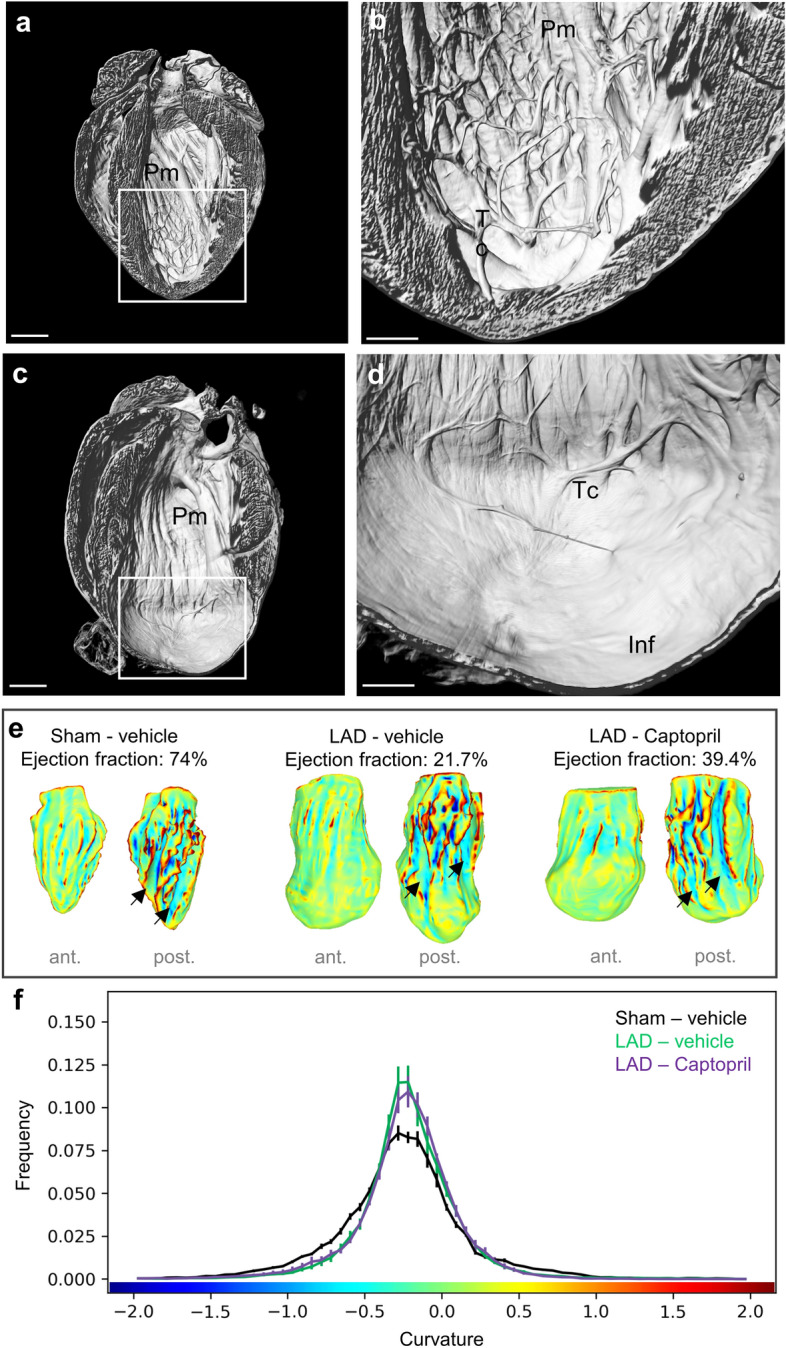
Light sheet imaging of left ventricle chamber topology. (**a**) Surface rendering of sham-operated light sheet imaged heart. (**b**) Magnified image demonstrates high optical resolution, intact papillary muscles (Pm) and dense distribution of trabeculae carneae (Tc). (**c**) Surface rendering of LAD-ligated light sheet imaged heart. (**d**) Magnified image demonstrates MI-induced changes in left ventricle (LV) chamber. Loss of trabeculae carneae (Tc) is evident in the dilated infarct area (Inf). (**e**) LV chamber surface is color-coded, with blue colours denoting concave surface and red convex surface areas (ant.—anterior view; post.—posterior view). Green indicates smooth surfaces. Outward dilation of the LV chamber is visible in LAD ligated hearts. Indentations left by papillary muscles are indicated by arrows. Lack of curvature is visible in the posterior view of the LAD—vehicle heart, with papillary muscle and trabecular indentations absent from the apex. Ejection fraction (%) is shown for the selected hearts performed 5 weeks after surgery. (**f**) Quantitative analysis of LV chamber mean curvature. Analysis was carried out in the lower apical half the LV chamber and the group mean value is shown on the frequency distribution histogram. Data is presented as mean ± s.e.m., n = 8–10. Scale bars: (**a**,**c**) 1 mm; (**b**,**d**) 300 µm.

We analysed in detail the morphological changes in infarcted hearts. 3D reconstruction of light sheet microscopy-imaged hearts revealed intricate details in the distribution of papillary muscles, chordae tendinae and trabeculae carneae in the LV chamber (Fig. 4a,b). LAD ligated infarcted hearts demonstrated a thin LV wall and loss of trabeculae carnae in the dilated infarct zone 5 weeks post-MI (Fig. 4c,d). These results demonstrate that despite resistance to mortality LAD ligation results in extensive structural changes even in young mice. Since papillary muscles leave an imprint in the underlying LV chamber volume and their reorganization takes place in MI we asked whether there is a significant difference in the mean chamber surface curvature. In addition to papillary muscle changes the curvature is affected by dilation LV free wall. Of note, LV curvature analysis has been shown to reflect EF in patients with cardiovascular diseases^36,37^. Applying curvature analysis on light sheet imaging derived LV chamber volumes demonstrated a significant difference in the LV curvature distribution between LAD ligated and sham operated mice, with a curvature peak at -0.25 in LAD ligated groups corresponding to LV dilation (Fig. 4e,f). However, no significant differences were evident in LAD –Captopril group in comparison to LAD—vehicle group.

### Captopril improves border zone vascular density

**Figure 5:**
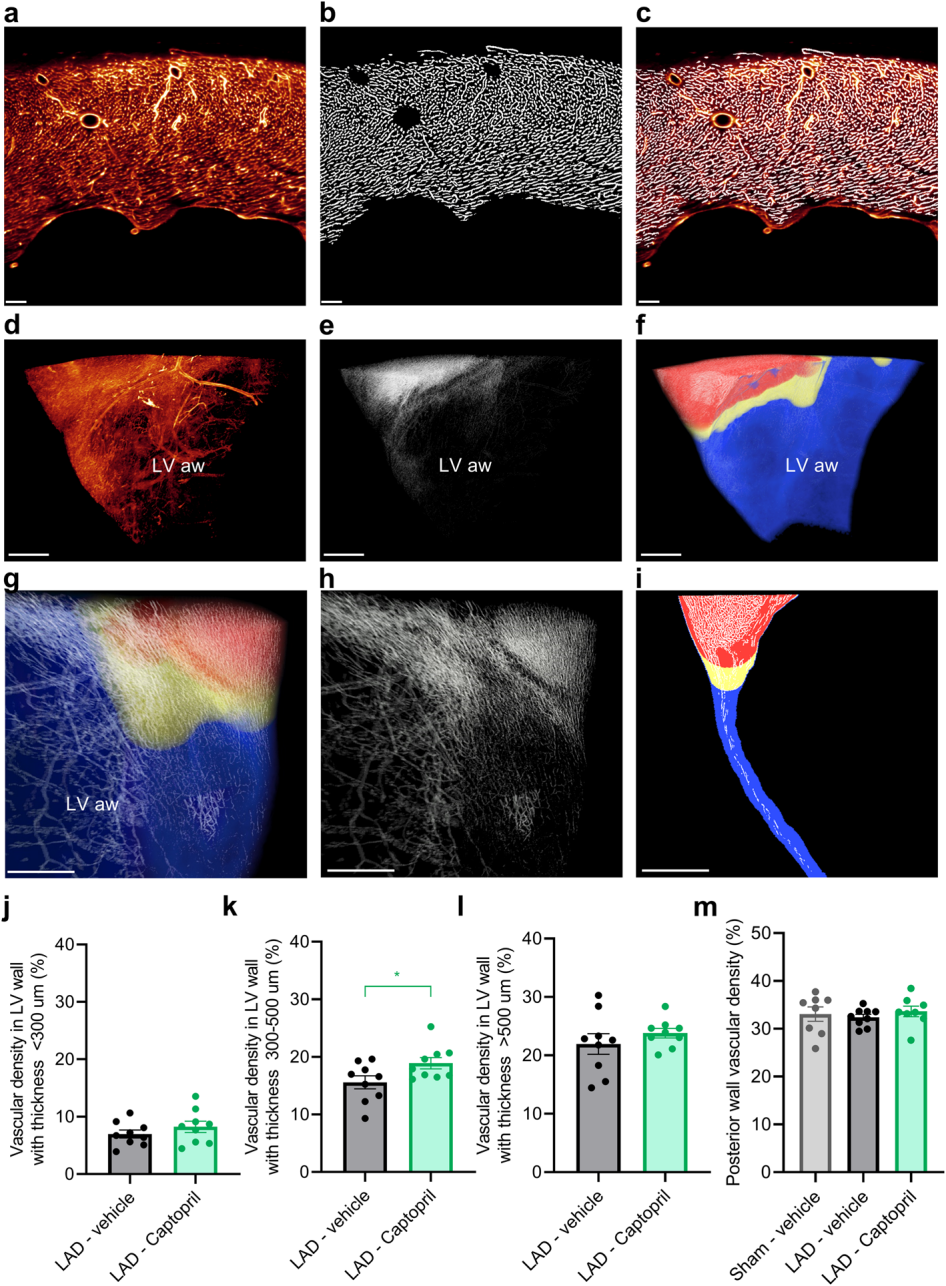
Captopril improves vascularization in the border zone. (**a**) 2D view of lectin perfused vasculature in the left ventricle (LV) wall. (**b**) Automatically segmented blood vessels in the same image. (**c**) Lectin perfused vasculature and overlay with automated detection (in white) of capillaries and arterioles. (**d**) 3D view of lectin perfused vasculature in the LV anterior wall (LV aw, view from the inside of the LV chamber towards the anterior wall). (**e**) Automated analysis of lectin signal in the same 3D image stack. (**f**) Wall thickness map corresponding to the same area. Thicker wall (> 500 µm) is shown in red, border zone with thickness between 300–500 µm in yellow and LV wall with thickness < 300 µm in blue. (**g-h**) Magnified view from the anterior side of LV. (**i**) 2D section through the LV wall, demonstrating wall thickness categories. (**j**) There is no difference in vascular density in the infarct zone (wall thickness < 300 µm) between vehicle and Captopril treated groups. (**k**) Captopril treatment improves vascular density in the border zone with wall thickness 300–500 µm. (**l**) No difference is visible between the groups in cardiac tissue with wall thickness above 500 µm. (**m**) Vascular density in posterior heart wall (no differences were observed between the study groups). One-way ANOVA with Tukey’s post hoc test. Significance: *p < 0.05. Data is presented as mean ± s.e.m., n = 8–9. Scale bars: (**a**–**c**), 100 µm; (**d**–**i**), 500 µm. LAD: left anterior descending artery ligation.

Since vascular density has a direct impact on cardiac function we focused on quantitative volumetric analysis of capillaries. To visualize blood vessels of the heart, mice were dosed intravenously with fluorescent DyLight649-tomato lectin (Fig. 5a). The infarcted LV and surrounding tissue were scanned at high resolution, enabling distinction of individual capillaries. A fully automated vessel detection algorithm for segmentation of the vasculature in 3D tissue volumes was utilized. The comparison of raw data of lectin labelled blood vessels and automated vessel detection algorithm demonstrated identification of microvascular capillaries and arterioles (Fig. 5a–c). Since vasculature analysis relies on in vivo lectin perfusion, the identified blood vessels are connected to functional circulation. Applying the algorithm on 3D scanned hearts made it possible to demonstrate vascular rarefaction in the infarct zone in the LV anterior wall (Fig. 5d,e).

Because of extensive and very dense capillary network small regional differences in microvascular patterning can remain undetectable in statistical analysis of the entire LV. For this reason, we developed an algorithm for automated 3D analysis of LV wall thickness to distinguish infarct zone with thin dilated LV wall (< 300 µm, blue), border zone (300–500 µm, yellow) and neighbouring myocardial tissue with healthy cardiomyocytes (above 500 µm, red). A 3D local thickness mask was generated for all samples (Fig. 5f), enabling visualization and acquisition of unbiased quantitative estimates on 3D distribution of blood vessels relative to LV wall thickness (Fig. 5g–i).

As expected, the infarct zone demonstrated low vascular density: 7 ± 0.66% of the total tissue volume in the LAD—vehicle compared to 8.2 ± 0.93% in the LAD—Captopril group (Fig. 5j, Supplementary Fig. 4a,b). In the border zone, vascular density was significantly higher in LAD—Captopril compared to LAD—vehicle (18.9 ± 0.93% versus 15.6 ± 1.08%, p < 0.05; Fig. 5k). In non-infarcted myocardium with wall thickness above 500 µm, no difference was found in vascular density (21.9 ± 1.02% in the LAD—vehicle group and 23.8 ± 0.76% in the LAD—Captopril group) (Fig. 5l). No statistically significant difference in vascular density was observed in LAD operated groups in comparison to sham group in the posterior LV wall (Fig. 5m). In the Sham—vehicle group the density was 33.1 ± 1.5%, in LAD—vehicle group 32.4 ± 0.6% and in LAD—Captopril group 33.6 ± 1.1%.

## Discussion

ACEi have remained the primary therapeutics for MI, yet the development of new drugs has been hampered by insufficient understanding of the molecular mechanisms of heart failure and the role existing drugs play at different periods in post-MI remodelling. In addition, difficulties in characterizing drug effects in animal models has slowed the translation of results from preclinical studies to patients. Here we developed a light sheet imaging method to provide detailed quantitative characteristics of myocardial infarction in mice and combined this with echocardiography to demonstrate the effects of captopril at late stages of post-MI remodelling.

Analysis of cardiac morphology in rodents has been challenging due to technical limitations. Standard echocardiography provides valuable information on cardiac function but lacks the resolution to chart smaller regional differences and the ability to characterize changes in coronary vasculature. Histological assessment of MI is on the other hand challenging due to irregularity of the infarct, thus requiring careful reconstruction of the sectioned heart. Here we addressed these limitations by implementing a light sheet microscopy approach, combined with automated image analysis. Light sheet fluorescence microscopy has emerged as a powerful tool for visualizing the morphology of different organs, the distribution of cell types and activation of signalling pathways. Clearing protocols have been developed for specific tissue types and organs enabling light sheet imaging of large transparent samples^38^. Recent years have witnessed the implementation of 3D light sheet imaging in cardiovascular research. It has been used to provide mechanistic insights into doxorubicin-induced cardiac injury in zebrafish larvae^39^. It has also been applied to visualize arterial injury response in mice and rats and to quantify plaque formation in mouse models of atherosclerosis^40–42^. Light sheet imaging has likewise been used for mouse hearts^43,44^ and a specialized optical clearing protocol has been developed enabling visualization of vascular reorganization and immune cell infiltration following ischemia reperfusion injury^45^. While light sheet imaging has been used to study the effect of pharmaceuticals in the brain^46,47^, it has yet to be used in preclinical cardiovascular pharmacological research. In order for 3D light sheet imaging to be applicable in drug discovery research the samples need to have highly reproducible morphological quality, but importantly for the heart be also fixed in the same phase of cardiac cycle. The hereby developed protocol combining cardioplegic solution for stopping hearts in diastole and rapid fixation in glyoxal for maintaining tissue integrity provided uniform sample quality and enabled the development of automated image analysis tools for unbiased data analysis. Light sheet imaging has limitations in cardiovascular research, arising mostly from the nature of cardiac physiology. A single heart can only be analysed in either systole and or diastole and thus some functional parameters cannot be directly assessed. Combining echocardiography and light sheet imaging offers a way to overcome these limitations and provide more accurate insights into cardiac function than either technology can alone.

Our data supports a positive effect of captopril on cardiac function following MI. The obtained effect on EF, amounting to 12.6% improvement in LAD ligated mice, is in line with improvements of ~ 8–9% in clinical trials as compared to baseline^48–51^. Ultrasound analysis of mouse and rat LAD-ligation models have revealed beneficial effects of captopril on cardiac hemodynamic parameters, both when treatment is started immediately after MI^52^ or delayed by one week^12^. Likewise, administration of ACEi imidapril from the onset of MI and over 4-week period improved diastolic function in rats^53^.

The mechanisms by which ACEi improve cardiac function are complex and still poorly characterized, largely because they affect multiple processes at different time points following MI. ACEi treatment is started early in clinical practice following MI, resulting in improved survival^54^. By implementing a delay in ACEi treatment enabled us to prevent its action on fibrotic scar formation and focus specifically on the question of whether and how ACEi affect the subsequent remodelling phase after the fibrotic infarct zone has been fully established. Young mice survive severe myocardial infarction thus allowing to dissect such late role of captopril. Data from echocardiography and light sheet imaging show that delayed captopril treatment does not affect the thickness of the LV anterior wall and reduce the size of infarcted myocardium, indicating that once the fibrotic scar is formed captopril treatment does not have a significant impact on its size. In contrast, 3D image analysis shows that delayed captopril treatment reduces LV dilation. Previously published analysis of LV wall dilation on histological sections from captopril treated mice failed to detect a positive effect^12^, whereas a study using lisinopril showed improvement on cardiac chamber morphology^55^. Large meta-analyses of clinical studies have demonstrated a positive effect of early ACEi treatment on reducing LV dilation in clinical settings^56^. Our results also suggest a positive effect of captopril on reducing cardiac hypertrophy, which is evident in reduced heart weight and posterior LV wall thickness, in line with previous findings^57–59^. In sum, captopril has a delayed effect on preventing cardiac hypertrophy and LV wall dilation.

Our data from 3D imaging shows that delayed captopril treatment leads to higher vascular density in the infarcted heart border zone but has neither positive nor negative effect on vascular density in the infarct zone and in the posterior cardiac wall. Still little is known about the dynamic response of microvasculature to MI, although angiogenesis has emerged as a lucrative target in pharmacological research^15,16,60^. Captopril has been reported to inhibits angiogenesis in tumours^61^ and skeletal muscle^62,63^, but no significant effect has so far been found on the angiogenesis in the infarcted heart^57,64,65^. Considering the anti-angiogenic role of captopril, it is on one hand likely that the effect on vascular density that we observed in the border zone is due to general improvement in perfusion and in cardiac morphology rather than neovascularization. One the other hand, captopril has also been found to inhibit capillary degeneration in diabetic retinopathy^66^. It may have a similar role in the infarcted heart, preventing early loss of microvasculature following an infarct. A possible explanation could also involve captopril induced upregulation of IGF-1 (insulin-like growth factor 1) system in the infarct border zone^67^, which has known roles in promoting microvasculature remodelling and angiogenesis^68^. These results highlight localized effect of captopril in the infarct border zone.

Using correlative functional and light sheet imaging, this study provides detailed characterization of the effect of captopril on postinfarction remodelling in a mouse chronic heart failure model. Captopril helps to preserve cardiac morphology, limits LV wall dilation, improves border zone vascularisation and results in improved EF following MI.

## Supplementary Information

Supplementary Information.

Supplementary Video 1.

Supplementary Video 2.
