# Supplementary material for: Effect of captopril on post-infarction remodelling visualized by light sheet microscopy and echocardiography
Source: Sci Rep. 2021 Mar 4;11:5241. doi: 10.1038/s41598-021-84812-7 (PMC7933438; doi:10.1038/s41598-021-84812-7)

Effect of captopril on postinfarction remodelling visualized by light sheet microscopy and echocardiography

Urmas Roostalu[1*], Louise Thisted[1], Jacob Lercke Skytte[1], Casper Gravesen Salinas[1], Philip Juhl Pedersen[1], Jacob Hecksher-Sørensen[1], Bidda Rolin[1, 3], Henrik H. Hansen[1], James G Mackrell[2], Robert M Christie[2], Niels Vrang[1], Jacob Jelsing[1], Nora Elisabeth Zois[1]

1. Gubra, Hørsholm Kongevej 11, B, 2970 Hørsholm, Denmark

2. Lilly Research Laboratories, Eli Lilly and Company, Indianapolis, Indiana 46285, USA

3. Present address: Novo Nordisk, 2760 Maaloev, Denmark

*uro@gubra.dk

Supplementary material

Supplementary Figure 1. Comparison of fixative effect on cleared heart morphology. Hearts were immersion fixed in either glyoxal **(a-b, e)**, 4% paraformaldehyde (PFA, **c**) or 10% neutral buffered formalin (NBF, **d**). **(a)** 3D reconstructed overview of glyoxal fixed cleared mouse heart. **(b)** Section from the same heart in horizontal long axis plane. **(c-e)** section through the apical region of the heart (along short axis plane). Arrows on (c) and (d) indicate abnormal cardiac morphology (wavy, irregular surface). Arrowheads indicate autofluorescence of the blood in the left ventricle chamber. Blood remains brightly autoflorescent in PFA and NBF fixed hearts. Scale bars: 200 µm.

Supplementary Figure 2. Development of deep learning-based model for LV chamber segmentation. (a) 3D images from anterior and posterior view and 2D slice through the LV chamber. Manually validated semi-automated volume is shown in blue, fully automated deep-learning based segmentation in yellow. Overlay (white on 2D section) illustrates accuracy of LV chamber segmentation. **(b)** Training curve data demonstrates saturation of dice coefficient after ~40 epochs of training. Scale bars: 1 mm.

Supplementary Figure 3. Autofluorescence channel enables visualization of tissue architecture in the infarcted heart. Transition from healthy cardiac tissue to infarct is shown on 2D image plane, with magnified areas shown for selected regions (1-4). Cardiomyocytes can be detected by bright autofluorescence in the red wavelength (images 1-2), whereas fibrotic tissue that lacks cardiomyocytes can be distinguished by low level autofluorescence (images 3-4). Scale bar: 400 µm, in magnified images 200 µm.

Supplementary Figure 4. Vascular density analysis. (a) 3D image of computationally detected vasculature in a representative sample from LAD – vehicle group. **(b)** 3D image of computationally detected vasculature in a representative sample from LAD – Captopril group. Scale bars: 200 µm. LV: left ventricular.

Supplementary Movie 1. Light sheet imaging of sham operated mouse heart. Pseudocoloured view of tissue autofluorescence.

Supplementary Movie 2. Light sheet imaging of LAD-ligated mouse heart. Pseudocoloured view of tissue autofluorescence demonstrates the distribution of major blood vessels, suture on the ligated LAD and LV wall dilation in the infarct zone.

Supplementary Figure 1

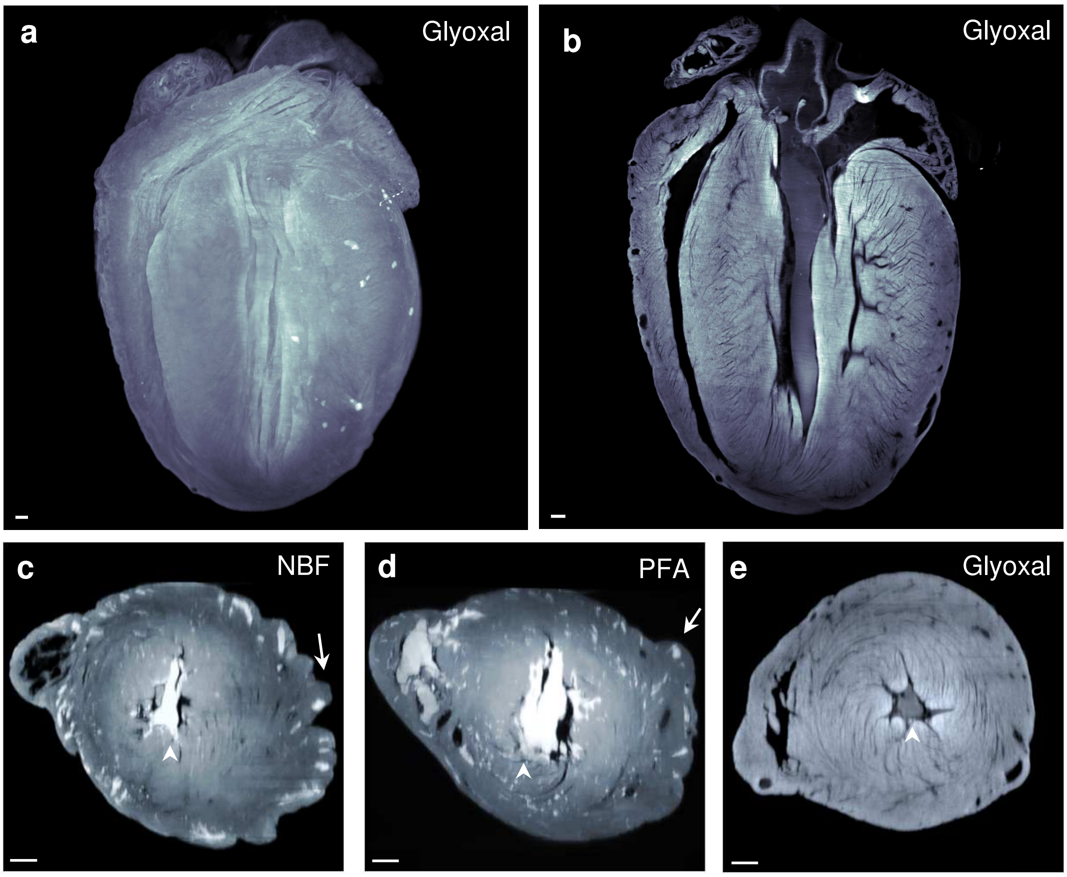

Supplementary Figure 2

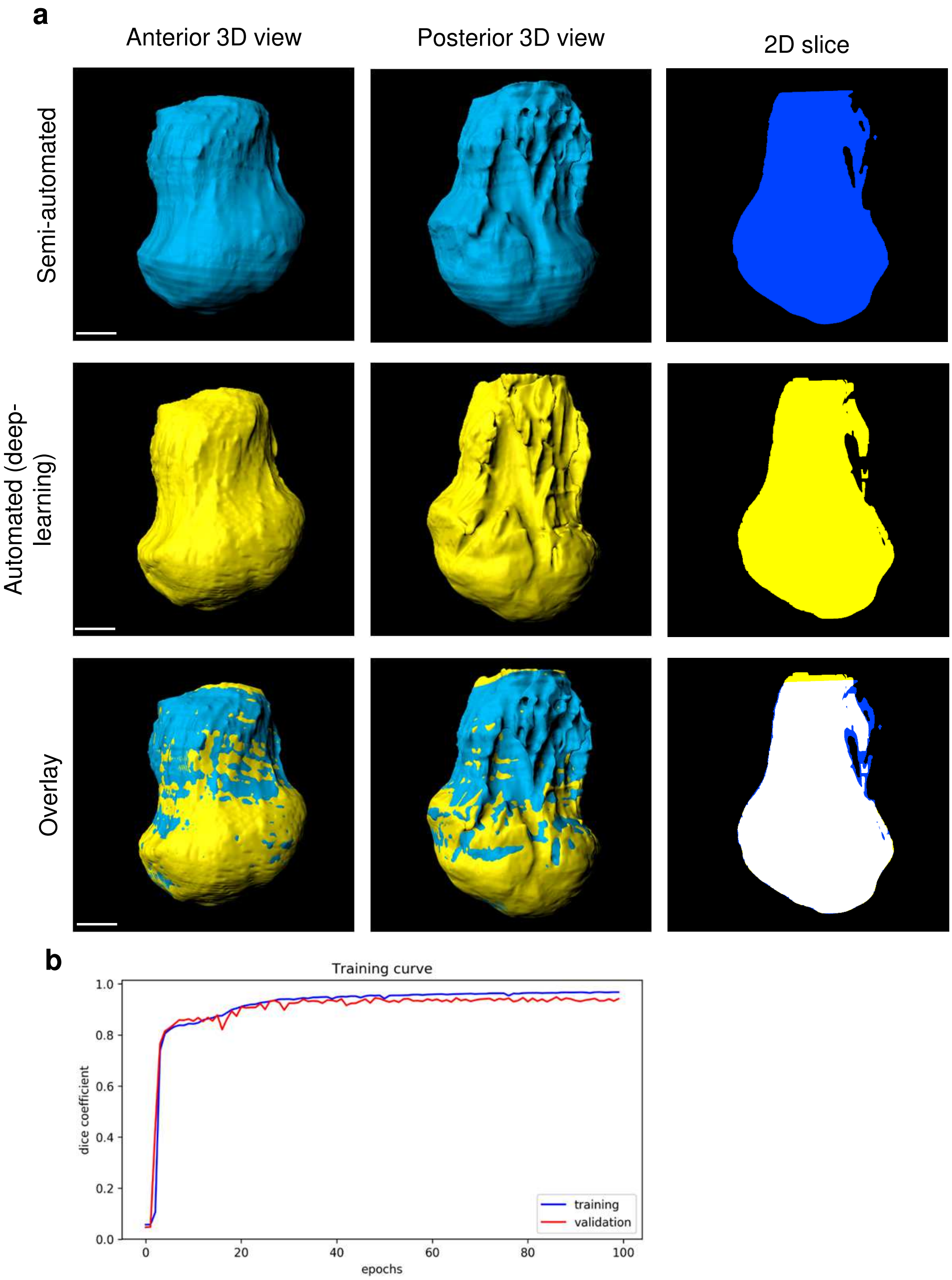

Supplementary Figure 3

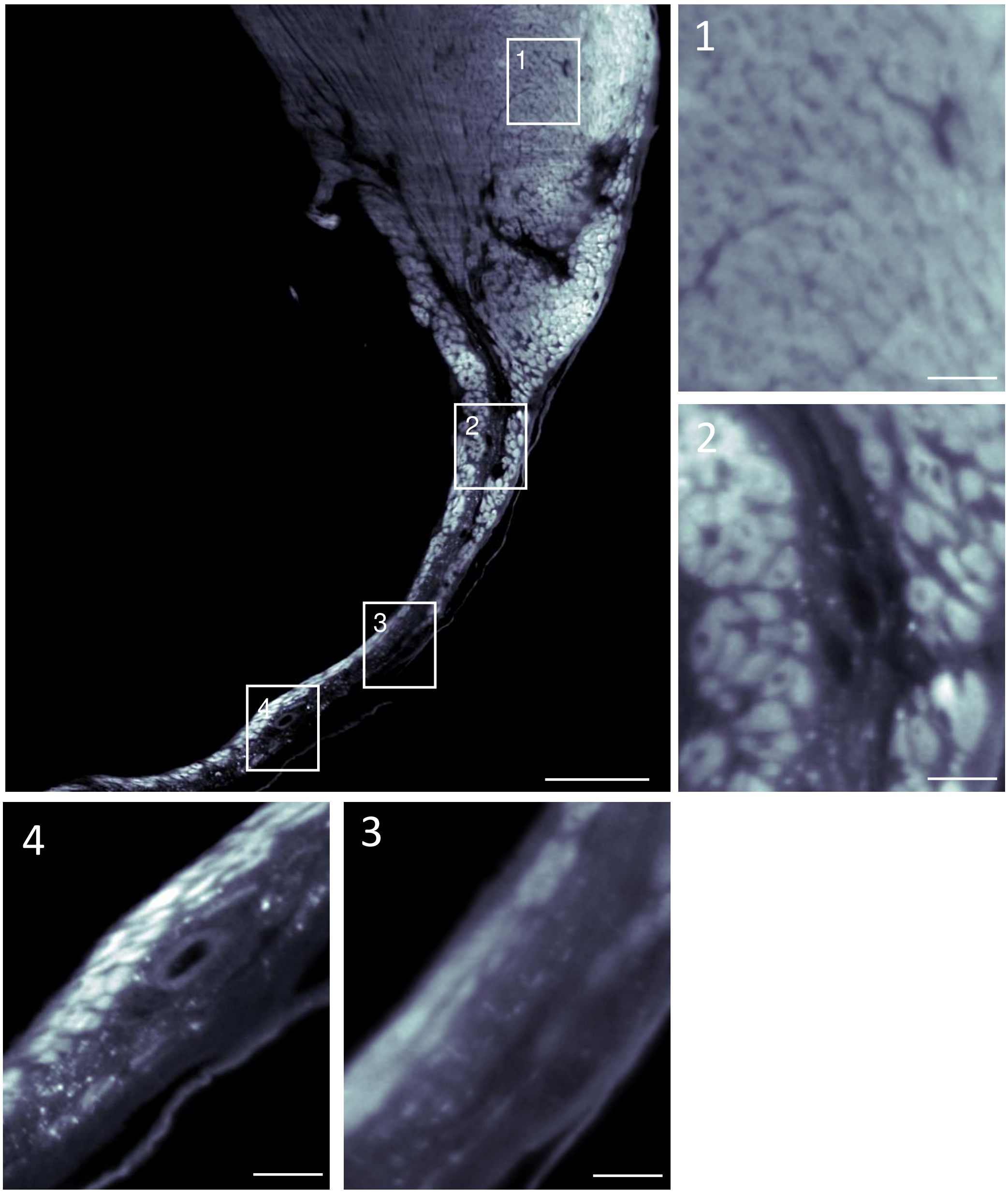

Supplementary Figure 4

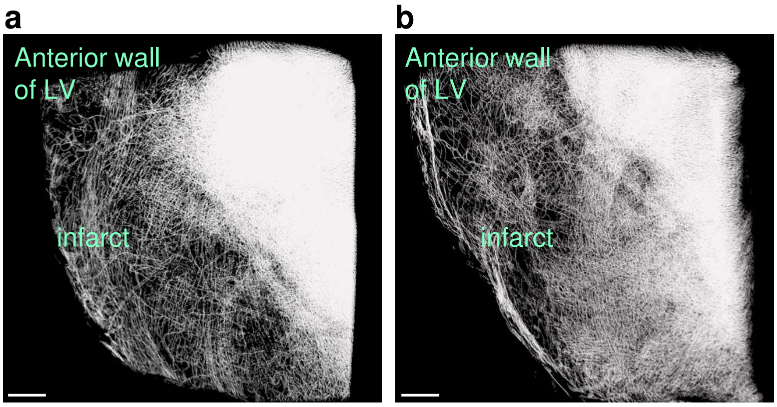

Supplement: Supplementary file 1 — Supplementary Information. [file 41598_2021_84812_MOESM1_ESM.pdf]
